# Patterns of Adverse Childhood Experiences and Postpartum Depressive Symptoms

**DOI:** 10.1007/s40653-025-00720-2

**Published:** 2025-06-14

**Authors:** Sunny H. Shin, Changyong Choi, Gabriela Ksinan Jiskrova, Camie A. Tomlinson, Casey B. Corso, Tiffany Kimbrough

**Affiliations:** 1 College of Public Health, Department of Social Work, George Mason University, 4408 Patriot Circle, Fairfax, VA 22030 USA; 2 Department of Social Welfare, Gachon University, Sujeong-gu, Seongnam-si, Gyeonggi-do Republic of Korea; 3 Research Centre for Toxic Compounds in the Environment, Faculty of Science, Masaryk University, Brno, Czech Republic; 4 Kent School of Social Work and Family Science, University of Louisville, Louisville, KY USA; 5 Department of Psychology, Virginia Commonwealth University, Richmond, Virginia, USA; 6 School of Medicine, Department of Pediatrics, Virginia Commonwealth University, Richmond, Virginia, USA

**Keywords:** Adverse childhood experiences, Postpartum depression, Maternal health, Depression screening, Latent class analysis

## Abstract

Adverse childhood experiences (ACEs) have been linked with increased risk for postpartum depression, which subsequently can lead to poor maternal and infant outcomes. The present study investigated how different patterns of ACEs are associated with postpartum depressive symptoms and with use of depression screening services. A racially/ethnically diverse sample of low-income women (*N* = 427) in an urban, university hospital in the Mid-Atlantic region reported their ACEs, depressive symptoms, receipt of depression screening, and receipt of a postpartum home visit. Three latent classes of maternal ACEs were identified: Low ACEs (57% of the sample), High Parental Separation/Divorce (30%), and High/Multiple ACEs (13%). Participants in the High/Multiple ACEs classes reported the highest levels of depressive symptoms, followed by women in the High Parental Separation/Divorce class, then the Low ACEs class. There were no statistically significant differences in depression screening services used across the three classes. Findings highlight the importance of screening for maternal ACEs during the perinatal period and targeting depression prevention services based on ACEs. More specifically, findings suggest multiple types of ACEs at high levels may be a more important predictor of depressive symptoms postpartum than the specific types of ACEs that are experienced.

Postpartum depression (PPD), depression occurring after childbirth and typically lasting up to a year, is a common and significant public health issue. During the first six-months postpartum, 13.2% of women experience depressive symptoms (Bauman et al., 2020), which have been associated with a wide variety of negative maternal and child health outcomes, disrupted maternal-child bonding, and long-term cognitive, emotional, and behavioral development problems in children (Chapman & Wu, 2013; Dagher et al., 2021; Meaney, 2018; Slomian et al., 2019). Further, the cost of untreated perinatal mood and anxiety disorders (which includes PPD) is approximately $14 billion across the first five years postpartum (Luca et al., 2020).

Given the tremendous public health and economic burden of perinatal mood and anxiety symptoms, there is a growing consensus on the importance of screening for PPD symptoms (Selix & Goyal, 2018). Screenings can lead to timely treatment referrals, mitigating detrimental effects of PPD. In this regard, clinical professionals, such as the American Academy of Pediatrics (Earls et al., 2019), American College of Obstetricians and Gynecologists, (2018), and U.S. Preventive Services Task Force (Siu et al., 2016), consistently recommend screening for maternal depression during prenatal, postnatal, and routine pediatric examinations. A recent Pregnancy Risk Assessment Monitoring System (PRAMS) report indicated that a higher proportion of women were asked about their depressive symptoms during prenatal (79.1%) or postpartum (87.4%) visits (Bauman et al., 2020). Other studies, however, show that a significant proportion of women are not screened for depression during perinatal visits. For example, Cox et al. (2016) indicated that over half of women with perinatal depression are undetected or undiagnosed. Therefore, it is an important public health task to identify risk factors of PPD. Identifying women who may be most at risk can inform screening, prevention, and intervention strategies.

## Adverse Childhood Experiences and PPD Risk

Although the association between exposure to violence and PPD symptoms has been examined for several decades (see Alvarez-Segura et al. (2014), for a review), researchers have more recently focused on the association between adverse childhood experiences (ACEs; i.e., potentially traumatic events that occur in childhood (Centers for Disease Control and Prevention, 2019; Felitti et al., 1998) and PPD symptoms (Folger et al., 2018; Menke et al., 2019; Morrison et al., 2017). ACEs have been linked to long-term physical, mental, and behavioral health challenges (Bellis et al., 2019). Research has shown that ACEs contribute to heightened vulnerability to depression, anxiety, and PTSD, often persisting into adulthood and exacerbating perinatal mental health challenges (Hughes et al., 2017; Racine et al., 2021) For example, individuals with high ACEs exposure face increased risks for chronic mental health problems and mood disorders, such as PPD, due to the cumulative effects of early life stress on emotional regulation and neurobiological functioning (McLaughlin et al., 2010; Nurius et al., 2015). A recent meta-analysis of seven 2018–2020 studies found that maternal ACEs were positively associated with PPD symptoms (*r* =.23; Racine et al. (2021).

Due to the frequent co-occurrence of ACEs (Atzl et al., 2019; Hemady et al., 2021; Jasthi et al., 2021; Kim et al., 2020; Menke et al., 2019; Osofsky et al., 2021) and potential synergistic interactions among different types of ACEs that increase risk for maladaptive outcomes (Briggs et al., 2021; Hughes et al., 2017), person-centered approaches that can capture underlying complex patterns of ACEs are warranted. Latent class analysis (LCA) is one specific person-centered approach that can be used to define underlying subgroups (i.e., classes) of individuals based on their common response patterns to specific indicators, such as different types of ACEs (Masyn, 2013). Researchers utilizing LCA can also examine the effects of predictors on latent class membership and how latent class membership predicts distal outcomes (Nylund-Gibson et al., 2019; Nylund-Gibson & Choi, 2018). Thus, this approach can provide a more nuanced examination of the relation between exposure to ACEs and PPD.

A limitation of previous studies is the predominant examination of the dosage effect of ACEs on PPD by using a count of different types of ACEs (i.e., cumulative ACEs scores). The cumulative score approach limits researchers in that it assumes all types of ACEs have equal weight on study outcomes through the creation of the sum score (Finkelhor et al., 2013; Lanier et al., 2018). Recent research has expanded on the cumulative score method by characterizing subgroups of mothers whose ACEs are qualitatively similar to each other (Atzl et al., 2019; Merrick et al., 2020; Osofsky et al., 2021). However, to our knowledge, only two published studies with U.S. samples utilized LCA of maternal ACEs in relation to PPD (Nidey et al., 2020; Stargel & Easterbrooks, 2020), both of which identified a four-class model characterized by: (a) low exposure to ACEs, (b) high exposure to multiple ACEs, (c) high exposure to household dysfunction, and (d) high exposure to child maltreatment. These studies provide evidence that identification of ACEs patterns may provide more nuanced and specific information regarding women who may be most at risk of PPD symptoms, which can critically inform PPD intervention and prevention efforts.

Although extant literature evidences that maternal exposure to ACEs increases risk for PPD symptoms, there is limited research that has utilized person-centered approaches to identify groups of women characterized by unique patterns of ACEs exposure. Therefore, the present study had three aims: (a) to identify latent classes of women based on their patterns of exposure to ACEs, (b) to examine the associations between latent class membership and PPD symptoms, adjusting for the effects of covariates, and (c) to determine whether latent class membership is associated with PPD screening. This approach provides a more nuanced, dimensional method of examining ACEs and builds on the current literature regarding the detrimental effects of maternal ACEs on PPD.

## Materials and Methods

The present study utilized a sample of 427 women at a large university medical center who participated in the Longitudinal Infant and Family Environment (LIFE) study. The LIFE study was designed to examine the impact of a hospital-based intervention to promote safe infant sleep practices. Consenting, English speaking women over age 18 completed a baseline survey prior to receiving the intervention at the hospital. The Institutional Review Board (IRB) of Virginia Commonwealth University reviewed and approved the study procedures. A detailed description of the intervention can be found in a previous study (Shin et al., 2019). Follow-up data were collected around seven days via nurses at home visitation and three months postpartum via online survey.

### Measures

#### Adverse Childhood Experiences

Participants retrospectively reported their exposure to ACEs before age 18 during the postpartum home visit. Participants responded whether they had been exposed (1) or not (0) to each of the traditional ACEs items (Felitti et al., 1998).

#### Postpartum Depressive Symptoms

PPD symptoms were measured using the modified version of the Patient Health Questionnaire-2 (PHQ-2), which was adapted by the CDC’s PRAMS study (Bauman et al., 2020). The items were rated on a 5-point Likert-type scale, ranging from *never* (0) to *always* (4), and then summed. The PHQ-2 has been validated as an effective screening tool for postpartum depression, demonstrating high sensitivity and specificity within pregnant and general adult populations (Chae et al., 2012; Löwe et al., 2005; Smith et al., 2010).

#### Screening for Postpartum Depression

Screening for PPD was assessed at the three-month follow-up by the following two items: “Since your new baby was born, have you had a postpartum checkup for yourself?” and “During your postpartum checkup, did a doctor, nurse, or other health care worker ask if you were feeling down or depressed?” The items were answered *yes* (1) or *no* (0). Participants were also coded as having received a PPD screening (1) or not (0).

#### Demographic Information

Demographic information, collected at baseline, included maternal age, race/ethnicity, educational attainment, family income, and infant birth order. Race/ethnicity was dichotomized due to sample distribution (White/non-Hispanic individuals = 1; racial/ethnic minority individuals = 0). Similarly, educational attainment was dichotomized (less than high school or high school degree or less = 0; greater than high school degree = 1). Family income, age and infant birth order were treated as continuous variables.

### Analysis

Latent class analysis (LCA) was used to identify distinct ACEs exposure classes. The optimal number of classes was evaluated by comparing model fit indices, the proportion of participants in the classes, and theoretical meaningfulness of the classes (Nylund-Gibson & Choi, 2018). Akaike Information Criterion (AIC), Bayesian Information Criterion (BIC), a sample-size adjusted BIC, Lo-Mendell-Rubin Adjusted Likelihood Ratio Test (LMRT), and Bootstrapped Likelihood-Ratio Test (BLRT) were used to evaluate the model fit. Lower values of AIC, BIC, and adjusted BIC indicate better model fit and statistically significant LMRT and BLRT indicate that a model with one additional class fits significantly better in comparison to a model with one less class (Nylund et al., 2007; Nylund-Gibson & Choi, 2018). Further, higher entropy values (range: 0–1) indicate higher classification certainty (Nylund et al., 2007).

The associations between ACEs classes and two distal outcomes (i.e., PPD symptoms and screening for PPD) were examined using the maximum likelihood (ML) three-step approach (Asparouhov & Muthén, 2014; Nylund-Gibson et al., 2019). Once the best-fitting LCA model was selected, a new data set containing most likely class membership assignments from the model was saved. Wald tests (Asparouhov & Muthén, 2014) were used to determine the statistical significance of the differences in class-specific intercepts for depressive symptoms and screening for PPD. In the third step, posterior distributions found through the first step were adjusted and covariate effects on class membership were controlled for and relations between class membership and distal outcomes were examined. All data analyses were performed using Mplus 8.0 software (Muthén & Muthén, 2017).

## Results

A summary of descriptive statistics is provided in Table 1. Mean maternal age was 26.5 years and 50.1% of participants were single. Majority of the study population was low-income (97.4%, less than $50,000 household annual income), racial/ethnic minorities (70.0% of non-Hispanic Black, 13.1% of Hispanic, and 4.4% Other), and had low education attainment (88.1%, high school/GED or less). 62.5% of the study participants were exposed to at least one ACE, 15.0% were exposed to four or more ACEs, and on average were exposed to 1.6 ACEs. ACE prevalence ranged from 3.6% (sexual abuse) to 59.1% (parental separation/divorce). The modified PHQ-2 mean score was 1.75. Three-month follow-up participants reported that they were screened for depression during postpartum check-ups, though a quarter of participants had not completed a postpartum check-up (*n* = 41) or been asked for their depressive symptoms during postpartum check-up(s) (*n* = 9).

**Table 1:** Descriptive statistics of the study variables

Variable	*n*	Valid %/ Mean (SD)
Maternal age	427	26.5 (5.8)
Maternal race/ethnicity		
African American	299	70.0
Non-Hispanic White	53	12.4
Hispanic	56	13.1
Asian	4	0.9
Other	15	3.5
Educational attainment		
≤ high school or GED	376	88.1
> high school or GED	51	11.9
Marital status		
Married or cohabiting	212	49.9
Never married, separated, divorced, or widowed	213	50.1
Annual Household income		
Under $25,000	316	74.4
$25,000– $39,999	83	19.5
$40,000– $49,999	15	3.5
$50,000– $74,999	4	0.9
$75,000– $99,999	4	0.9
Over $100,000	3	0.7
Birth order of the infant	426	1.9 (1.1)
Adverse childhood experiences ^a^		
Emotional abuse	66	15.6
Physical abuse	45	10.7
Sexual abuse	15	3.6
Emotional neglect	61	14.5
Physical neglect	24	5.7
Parental separation/divorce	251	59.1
Intimate partner violence exposure	34	8.2
Household alcohol/drug abuse	89	21.2
Household mental illness	42	10.0
Household incarceration	77	18.4
Depressive symptoms	426	1.8 (1.6)
Postpartum checkup ^a^	157	79.7
Screening for postpartum depression ^a, b^	146	93.0

Note. GED = General Educational Development

^a^*n* and valid percentage of participants who answered affirmatively to the items

^b^*n* and valid percentage of participants who received a postpartum checkup

**Fig. 1:**
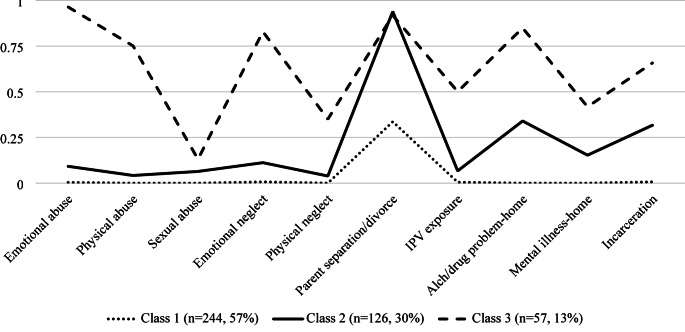
Item-Response Probabilities for 10 ACEs Across the Three Classes. *Note.* Class 1 = Low ACEs; Class 2 = High Parental Separation/Divorce; Class 3 = High/Multiple ACEs

Fit indices, displayed in Table 2, suggested either a two-, three-, or four-class solution as acceptable. Considering the AIC, BIC, aBIC, BLRT, theoretical meaningfulness of the classes, and the number of individuals in the classes (> 5–8% of the sample; Nylund-Gibson and Choi (2018), the three-class solution demonstrated the best fit. While the four-class solution had the lowest AIC and BIC, it included a class with only 4% of the sample, raising concerns about stability and interpretability. The three-class model retained adequate class separation (entropy = 0.79), and all class sizes exceeded 10%, supporting its practical and theoretical utility. In contrast, the LMRT was not significant for the three- vs. two-class comparison (*p* =.08), but the BLRT remained significant (*p* <.001), suggesting incremental model improvement. Overall, the three-class solution provided the best balance between statistical fit, parsimony, and interpretability. Figure 1 depicts the item-response probabilities for each of the ACEs for the selected three-class model. In categorizing conditional probabilities as low (0–35%), moderate (35–50%), and high (50–100%), the current study aimed to reflect meaningful distinctions observed in our data.

**Table 2:** Fit statistics for the unconditional latent class analysis for 1–4 classes

*N*	AIC	BIC	Adjusted BIC	Entropy	LMRT	BLRT	Class probability
1	3250.40	3290.97	3259.24	N.A.	N.A.	N.A.	1.00
2	2515.83	2601.02	2534.38	0.94	*p* <.001	*p* <.001	0.82 / 0.18
3	2431.70	2561.52	2459.97	0.79	*p* =.08	*p* <.001	0.64 / 0.22 / 0.14
4	2424.71	2599.15	2462.70	0.81	*p* =.07	*p* =.01	0.64 / 0.21 / 0.12 / 0.04

Note. *N* = Number of classes; AIC = Akaike information criterion; BIC = Bayesian information criterion; LMRT = Lo-Mendell-Rubin Likelihood Ratio test; BLRT = Bootstrap Likelihood Ratio Test

Class 1 was characterized by low probabilities (< 0.001 − 0.336) of exposure to all ACEs. We labeled this class as *Low ACEs* (57% of the sample). Class 2 (30% of the sample) had a high probability of exposure to parental separation or divorce (0.939) and low probabilities of exposure to all other ACEs (0.039 − 0.317). Although parental separation/divorce was common in the overall sample (59.1%), its concentration within this class, combined with minimal exposure to other adversities, suggests a distinct and meaningful pattern of ACEs. This class was labeled *High Parental Separation/Divorce*. Lastly, class 3 was labeled *High/Multiple ACEs* (13% of the sample). This class had a high probability of exposure to seven ACEs (0.501 − 0.964) and a moderate probability of exposure to two ACEs, namely physical neglect (0.350) and family mental illness (0.418). The associations between ACEs class memberships and distal outcomes are summarized in Table 3. Two separate models for depression symptoms and screening for PPD were estimated via the ML three-step approach, controlling for maternal and infant demographic variables. After controlling for covariates, a significant link between ACEs classes and depression symptoms (χ^2^_(2, 424)_ = 20.97, *p* <.001) was found. Compared to women in the *Low ACEs* class (mean (*M*) = 1.19), those in the *High Parental Separation/Divorce* class (*M* = 1.83) and *High Multiple ACEs* class (*M* = 2.61) reported significantly greater PPD symptoms. Moreover, participants in the *High Multiple ACEs* class demonstrated significantly greater depressive symptoms than those in the *High Parental Separation/Divorce* class. Despite higher depressive symptoms of women in a riskier ACEs class, no statistically significant differences in screening for depression were found across the three classes (χ^2^_(2, 424)_ = 1.52, *p* =.467).

**Table 3:** Class-Specific intercepts for distal outcomes based on adverse childhood experiences classes controlling for covariates

	Class 1: Low ACEs			Class 2: High Parental Separation/Divorce			Class 3: High Multiple ACEs			Wald
	M	SE	d	M	SE	d	M	SE	d	
Depressive symptoms	1.19^C2, C3^	0.26	—	1.83^C1, C3^	0.35	0.12	2.61^C1, C2^	0.40	0.24	20.97^***^
Screening for PPD	0.72	0.12	—	0.68	0.13	0.02	0.57	0.16	0.06	1.52

Note. Superscripts refer to statistically different classes. The levels of statistical significance are provided in the text. C1 = Class 1; C2 = Class 2; C3 = Class 3; PPD = postpartum depression. PPD = postpartum depression. *d* = Cohen’s d. *p <.05, ^***^p <.001

Additionally, results showed an effect of several demographic variables on the likelihood of membership in the different ACEs classes. Compared to the *Low ACEs* class, being non-Hispanic Black was associated with a greater likelihood of membership in the *High Parental Separation/Divorce* class, (Odds ratio (*OR*) = 6.59, *p* <.001) while identifying as non-Hispanic White (*OR* = 47.61, *p* =.023) was associated with a greater likelihood of being in the *High/Multiple ACEs* class. Furthermore, a higher infant birth order (*OR* = 1.57, *p* =.001) was associated with a greater likelihood of being in the *High/Multiple ACEs* class, relative to the *Low ACEs* class. Finally, none of the covariates, aside from post-secondary education, were statistically significant in the main models. Post-secondary education was significantly associated with lower levels of postpartum depressive (PPD) symptoms, such that individuals with post-secondary education reported fewer symptoms (b = -0.57, *p* =.044). However, post-secondary education was not significantly associated with the likelihood of being screened for PPD.

## Discussion

The present study illuminates the relationship between maternal ACEs and PPD. We found that maternal ACEs patterns were differently associated with PPD symptoms, such that those with high levels of exposure to ACEs (i.e., high parental separation/divorce and high/multiple ACEs) reported significantly higher levels of depressive symptoms in comparison to those exposed to low ACEs. Those exposed to high/multiple ACEs reported the highest rates of depressive symptoms. There were, however, no significant differences in PPD screening by latent class membership. Additionally, race/ethnicity and birth order were significantly associated with class membership, though maternal education was the only covariate associated with PPD symptoms.

The present findings extend the literature by demonstrating that patterns of ACEs may differentially affect mental health during pregnancy and the postpartum period. Our results corroborate previous studies that found high exposure to ACEs to be associated with high levels of PPD (Nidey et al., 2020; Stargel & Easterbrooks, 2020). The current findings also align with results from cumulative risk literature. Still, our findings suggest screening for more nuanced ACEs profiles, rather than cumulative measures alone, are critical for more effective PPD screening and thus are likely to be more beneficial in helping ameliorate negative outcomes associated with PPD. Although recent studies have used LCA to examine maternal ACEs in relation to PPD (Racine et al., 2020; SmithBattle et al., 2021), our study extends this work in several important ways. Prior studies focused on specific subpopulations (i.e., adolescent mothers or participants in a home visiting program) while we used a community sample of young adult women, improving the generalizability of findings. Additionally, the present study examined both symptoms of PPD and screening for PPD, providing a more comprehensive picture of how maternal ACEs relate to both mental health and service engagement in the early postpartum period.

However, it is possible that exposures to multiple types of ACEs at high levels are a more important predictor of PPD symptoms than the specific types of ACEs that are experienced. This may be due to stress sensitization that occurs as a result of frequent exposure to external stressors, such as ACEs (McLaughlin et al., 2010; Nurius et al., 2015; Rousson et al., 2020). According to the stress sensitization model, exposure to multiple and/or repeated ACEs lowers the threshold necessary for future stressors to trigger symptoms of mental disorders, such as major depressive disorder (Stroud, 2020). For women with exposure to multiple and/or repeated ACEs, this sensitization places them at increased risk for PPD and other mental disorders. Exposure to early life stress, such as ACEs, has been shown to alter the regulation of cortisol, the primary stress hormone, resulting in either hyper- or hypo-responsiveness to stress in adulthood (Berens et al., 2017). Consequently, exposure to multiple types of ACEs may increase vulnerability to PPD through disruptions in stress-related neuroendocrine circuits (Maguire, 2019; Seth et al., 2016). These alterations can persist over time, leading to dysregulated stress responses during the perinatal period.

Women who have experienced multiple ACEs may also be at increased risk for PPD and depressive symptoms during the postpartum period due to re-traumatization upon recall of their own childhood experiences and memories (Meltzer-Brody et al., 2018; Oosterman et al., 2019). Further, women who have experienced ACEs may lack social support (e.g., family support), which is a key promotive factor for mental health during the transition to motherhood (Brown et al., 2012; Corrigan et al., 2015; Milgrom et al., 2019). Screening for ACEs is, therefore, an important aspect of perinatal care as this study and prior research indicates that exposure to ACEs increases women’s risk for PPD and other deleterious mental health outcomes. Future studies would benefit from considering a broader conceptualization of ACEs by measuring the frequency, duration, and timing of maternal ACEs to better understand what aspects of maternal ACEs places women at higher risk for poor mental health. Additionally, future studies should explore how prior ACEs may interact with adult stressors (e.g., exposure to intimate partner violence, racism, job loss) to further test the stress sensitization theory among pregnant and postpartum women.

We found no significant differences in screening for PPD based on patterns of ACEs. Still, our results add to the evidence that screening for maternal ACEs can help to identify women who may be at higher risk for PPD symptoms during the perinatal period and for whom PPD screening should be prioritized (Association of Women’s Health, Obstetric and Neonatal Nurses, 2015; Earls et al., 2019; Lewis Johnson et al., 2020). For example, building on the evidence that exposure to ACEs increases risk for PPD symptoms (Atzl et al., 2019; McDonald et al., 2019; Osofsky et al., 2021), individuals who report high exposure to ACEs should be flagged to ensure that they receive screening for depressive symptoms and PPD throughout the perinatal period. This approach can target women who may benefit the most from more thorough mental health assessment and timely treatment. Generally, it is recommended that women be offered multiple types of resources including formal support, such as psychiatric or therapeutic care, and informal resources including meditation and physical exercise to ensure the health and wellbeing of both the mother and infant (Ford et al., 2019; Olsen et al., 2021). These factors should be further examined to identify potential targets for PPD intervention and prevention efforts.

Interpretation of results should consider limitations of the current study. First, maternal ACEs were measured by retrospective binary self-report. While retrospective self-reports of ACEs of adult respondents have relatively high test-retest reliability (Dube et al., 2004; Mersky et al., 2017; Pinto et al., 2014), recall bias may be present. Further, frequency, severity, or duration of ACEs were not considered, and there may be other types of ACEs and adult stressful life events (e.g., intimate partner violence, racism) that may contribute to PPD risk that were unexplored. Maternal depressive symptoms were also measured by self-report and assessed in the early postpartum period in which some mothers may experience baby blues (i.e., fluctuation in mood, no interests, fatigue, anxiety). However, baby blues symptoms spontaneously resolve within 10 days (Langan & Goodbred, 2016) while PPD symptoms have been found to emerge early (Dennis, 2004) and persist up to 6-months postpartum (Kuo et al., 2014). Furthermore, only about half of the study population reported PPD screening experiences due to high attrition. While all participants were part of the LIFE study, which focused solely on infant sleep and did not include depression screening, the lack of significant differences in screening across ACEs-based classes may be more related to reduced power due to missing screening data. Finally, the generalizability of findings may also be limited by the characteristics of the sample, which was primarily composed of single, low-income, and racially/ethnically minoritized young women in an urban Mid-Atlantic setting. While this is a critically important population that is often underrepresented in research, findings may not extend to all postpartum populations. Additionally, there is potential for selection bias in that individuals who enrolled and remained in the study may differ meaningfully from those who did not participate or were lost to follow-up. For example, those with more severe PPD symptoms, unstable housing, or higher levels of adversity may have been less likely to participate or complete follow-up surveys, potentially underestimating the associations observed. Future studies should assess whether similar patterns are found in more demographically and socioeconomically diverse samples and consider strategies to reduce selection bias and improve retention.

## Data Availability

Not applicable.
